# A molecular approach for the rapid, selective and sensitive detection of *Exophiala jeanselmei* in environmental samples: development and performance assessment of a real-time PCR assay

**DOI:** 10.1007/s00253-015-7175-z

**Published:** 2015-11-28

**Authors:** X. Libert, C. Chasseur, A. Packeu, F. Bureau, N. H. Roosens, S. J. C. De Keersmaecker

**Affiliations:** Platform Biotechnology and Molecular Biology, Scientific Institute of Public Health (WIV-ISP), J. Wytsmanstraat 14, 1050 Brussels, Belgium; Health and Environment, Scientific Institute of Public Health (WIV-ISP), J. Wytsmanstraat 14, 1050 Brussels, Belgium; Mycology and Aerobiology, Scientific Institute of Public Health (WIV-ISP), J. Wytsmanstraat 14, 1050 Brussels, Belgium; Cellular and Molecular Immunology, Groupe Interdisciplinaire de Génoprotéomique Appliquée (GIGA), Université de Liège (ULg), Liège, Wallonia Belgium

**Keywords:** Black yeast, Detection, Environment, *Exophiala jeanselmei*, Real-time PCR, Molecular method

## Abstract

*Exophiala jeanselmei* is an opportunistic pathogenic black yeast growing in humid environments such as water reservoirs of air-conditioning systems. Because this fungal contaminant could be vaporized into the air and subsequently cause health problems, its monitoring is recommended. Currently, this monitoring is based on culture and microscopic identification which are complex, sometimes ambiguous and time-demanding, i.e., up to 21 days. Therefore, molecular, culture-independent methods could be more advantageous for the monitoring of *E. jeanselmei*. In this study, we developed a SYBR®green real-time PCR assay based on the internal transcribed spacer 2 from the 18S ribosomal DNA complex for the specific detection of *E. jeanselmei*. The selectivity (100 %), PCR efficiency (95.5 %), dynamic range and repeatability of this qPCR assay were subsequently evaluated. The limit of detection for this qPCR assay was determined to be 1 copy of genomic DNA of *E. jeanselmei*. Finally, water samples collected from cooling reservoirs were analyzed using this qPCR assay to deliver a proof of concept for the molecular detection of *E. jeanselmei* in environmental samples. The results obtained by molecular analysis were compared with those of classical methods (i.e., culture and microscopic identification) used in routine analysis and were 100 % matching. This comparison demonstrated that this SYBR®green qPCR assay can be used as a molecular alternative for monitoring and routine investigation of samples contaminated by *E. jeanselmei*, while eliminating the need for culturing and thereby considerably decreasing the required analysis time to 2 days.

## Introduction

*Exophiala* is a fungal genus containing numerous species recognized to be pathogenic (Nucci et al. 2001, 2002; Packeu et al. 2012; Woo et al. 2013a), and it is a member of the black yeasts. This genus is described as ubiquitous and has been isolated from diverse substrates (e.g., wood, soil, sludge, water, feed) (Dixon et al. 1980; Nishimura et al. 1987; Nucci et al. 2002). Within this genus, *Exophiala jeanselmei* is usually causing cutaneous or subcutaneous infections. However, *E. jeanselmei* infections are also frequently observed as a systemic infection or as a causal agent of cystic fibrosis in immunosuppressed patients (Nucci et al. 2001, 2002). Nevertheless, although limited data on the number of cases occurring are available and the epidemiology of airways infections caused by *E. jeanselmei* is poorly documented. Inside buildings, the way of contamination by this species is presumably linked to water reservoirs of air-conditioning units or pipings (Badali et al. 2012; Wang et al. 2001). As this species can cause health problems, its monitoring is needed.

Currently, monitoring of *E. jeanselmei* contamination of buildings is complicated and requires specific expertise. Indeed, in routine analysis, this monitoring is often based on culture, microscopic visualization, and visual counts (Anaissie et al. 2003). This approach depends on the growth of the culture, which for *E. jeanselmei* can take up to 21 days (Anaissie et al. 2003). In addition, this culturing is also influenced by the growth media chosen, the culture conditions such as temperature and humidity, or by competition between species (Pitkaranta et al. 2011; Vesper 2011). Moreover, *E. jeanselmei* is a complex species belonging to a group of morphologically difficult (or impossible) to differentiate species (Kawasaki et al. 2005; Zeng and De Hoog 2008; Zeng et al. 2007), i.e., the *Exophiala spinifera* clade (De Hoog et al. 2003), which groups among others *E. jeanselmei*, *Exophiala exophialae*, *Exophiala lecanii-corni*, and *Exophiala xenobiotica* (Kawasaki et al. 1999; Wang et al. 2001; Woo et al. 2013a; Zeng and De Hoog 2008)*.* Also, *Exophiala dermatitidis*, considered as an important and much studied pathogen inside the *Exophiala* genus, is morphologically similar to *E. jeanselmei* (Kawasaki et al. 1990, 2005; Masuda et al. 1989). A molecular approach could bring a solution to these bottlenecks in classification and identification of *E. jeanselmei* (Haase et al. 1995; Hee and Yoon 2002; Kawasaki et al. 1990).

Actually, the use of molecular tools (such as polymerase chain reaction (PCR), real-time polymerase chain reaction (qPCR) and sequencing) is being increasingly used for the detection, monitoring and the identification of fungi in general (Chemidlin Prevost-Boure et al. 2011; Hospodsky et al. 2010; Melkin et al. 2004; United States Environmental Protection Agency 2015). For example for the genus *Exophiala*, some PCR (Nagano et al. 2008; Najafzadeh et al. 2013; Sudhadham et al. 2010), qPCR and other molecular tools have been developed for the detection and identification of *E. dermatitidis* (Wang et al. 2013). Most often, these molecular tools target the 18S rDNA regions and especially the internal transcribed spacer 1 and 2 (ITS1 and ITS2). This ITS region is fully documented and a vast amount of data is available for in silico analysis. In 2012, Schoch and co-workers proposed to define these internal regions as a part of the barcode marker for fungi (Schoch et al. 2012). For *E. jeanselmei*, these ITS regions offer the possibility to develop molecular tools to discriminate the species, even inside the *E. spinifera* clade (Wang et al. 2001; Woo et al. 2013a; Zeng and De Hoog 2008). Despite these previous studies investigating the *E. jeanselmei* genotype (Badali et al. 2012; Kawasaki et al. 2005, 1999; Woo et al. 2013a), molecular tools for the specific detection of this fungus are however still poorly documented.

Based on ITS1 and ITS2, diagnostic molecular tools for *E. jeanselmei* infections were developed using PCR and classical Sanger sequencing (Nagano et al. 2008; Najafzadeh et al. 2013; Packeu et al. 2012; Wang et al. 2001; Woo et al. 2013b). But although these approaches have proven their effectiveness, they are not really adapted to routine analysis of many environmental samples due to the low specificity of the universal primers used in the PCR assay the time needed for the analysis and the cost. Indeed, environmental samples could contain more than one species. Then, in these samples, the use of universal primers would produce a mix of amplicons which would be poorly discriminated with Sanger sequencing. This problem could be solved by next-generation sequencing (NGS) tools but the associated costs and expertise needed (especially in data analysis) might be too high for routine analysis.

qPCR is an alternative approach offering specificity and a reduction in analysis time and cost, especially when many samples need to be analyzed. Also, a qPCR instrument might be more in the reach of a routine laboratory, as compared to a DNA sequencing instrument. Today, despite the development of qPCR tools for the detection of several fungal contaminants such as the monitoring of airborne molds from outdoor and indoor environment (Hospodsky et al. 2010; Libert et al. 2015; Nagano et al. 2008; Vesper 2011), no qPCR assay specific to *E. jeanselmei* yet exists.

In this context, this paper describes a SYBR®green qPCR assay for the specific detection of *E. jeanselmei*. To assess the performance of the developed qPCR assay, the selectivity, sensitivity, and efficiency were evaluated. As previously discussed (Libert et al. 2015), no harmonized guidelines exist for this performance assessment of qPCR assays for fungal detection. Therefore, we followed a similar approach as the one previously reported for *Aspergillus versicolor* (Libert et al. 2015) which is based on the guidelines developed for the qPCR detection of genetically modified organisms (GMO) (Broeders et al. 2014) and foodborne pathogens (Barbau-Piednoir et al. 2013) and the minimum information for publication of quantitative real-time PCR experiment (MIQE) proposed by Bustin et al. in 2009 (Bustin et al. 2009). Finally, a proof of concept for the detection of *E. jeanselmei* in environmental samples was provided, comparing the routine protocol based on classical techniques involving culturing steps, and the molecular qPCR method developed and evaluated in this study. Therefore, this study offers a new molecular tool based on SYBR®green chemistry which could be used as a routine protocol for the detection and/or the monitoring of *E. jeanselmei* in environmental samples, by all actors concerned.

## Materials and methods

### Fungal strains

Table 1 lists all the fungal species (*Acrenonium strictum*, *Alternaria alternata*, *Aspergillus fumigatus*, *Cladosporium cladosporioides*, *Cladosporium herbarum*, *Cladosporium sphaerospermum*, *E. dermatitidis*, *E. exophialae*, *E. jeanselmei*, *E. lecanii-corni*, *E. spinifera*, *E. xenobiotica*, *Penicillium chrysogenum*, *Stachybotrys chartarum*, and *Ulocladium botrytis*) and strains (19 strains in total) used in this study. They were all purchased from the BCCM/IHEM collection (Scientific Institute of Public Health in Brussels, Belgium).

**Table 1:** Selectivity evaluation of SYBR®green qPCR *Ejeanselmei*_*ITS* assay

Genus	Species	Reference BCCM/IHEM^a^	Positive signal	*C*_q_ mean ± SD	T_m_ mean ± SD (°C)
***Exophiala***	***jeanselmei***	**IHEM 4740**	**Yes**	**23.57 ± 0.38**	**79.75 ± 0.29**
*Exophiala*	*jeanselmei*	IHEM 4741	Yes	20.86 ± 0.22	79.25 ± 0.50
*Exophiala*	*jeanselmei*	IHEM 22665	Yes	21.81 ± 0.43	79.63 ± 0.25
*Exophiala*	*dermatitidis*	IHEM 9780	No	/	/
*Exophiala*	*exophialae*	IHEM 5976	No	/	/
*Exophiala*	*exophialae*	IHEM 20759	No	/	/
*Exophiala*	*lecanii-corni*	IHEM 3662	No	/	/
*Exophiala*	*spinifera*	IHEM 20752	Yes^b^	26.04 ± 0.05	78.50 ± 0.00
*Exophiala*	*xenobiotica*	IHEM 6582	No	/	/
*Acremonium*	*strictum*	IHEM 993	No	/	/
*Alternaria*	*alternata*	IHEM 4969	No	/	/
*Aspergillus*	*fumigatus*	IHEM 3562	No	/	/
*Cladosporium*	*cladosporioides*	IHEM 0859	No	/	/
*Cladosporium*	*herbarum*	IHEM 2268	No	/	/
*Cladosporium*	*sphaerospermum*	IHEM 1011	No	/	/
*Penicillium*	*chrysogenum*	IHEM 4151	No	/	/
*Penicillium*	*chrysogenum*	IHEM 20859	No	/	/
*Stachybotrys*	*chartarum*	IHEM 0359	No	/	/
*Ulocladium*	*botrytis*	IHEM 0328	No	/	/

The strain in bold is the reference used for the performance assessment and is fully characterized as *Exophiala jeanselmei* (IHEM 4740). Positive signal (Yes) is defined as an amplification with a C_q_ ≤ 40, and *T*_m_ value (°C) as expected. No defined as no amplification. C_q_ mean ± SD and T_m_ mean ± SD are based on two runs per extract from two independent DNA extracts for each strain which have given a positive signal in qPCR using 1000 theoretical genomic copies

^a^IHEM/BCCM collection, Mycology and Aerobiology, Scientific Institute for Public Health, rue Juliette Wytsman 14, 1050 Brussels, Belgium

^b^ *T*_*m*_ is different

### Culture conditions

The fungal strains were grown as described previously (Libert et al. 2015). Briefly, the fungal strains were spiked into a S10 Sabouraud liquid medium (Biorad, Temse, Belgium) and were grown at 25 °C with constant agitation between 3 to 21 days according to the species’ growth conditions.

### DNA extraction

DNA of the fungal cultures was prepared as previously reported (Libert et al. 2015). Briefly, after the incubation time, 300 mg of wet sample were transferred to cryotubes containing 0.25 ml of acid-washed glass beads (Sigma Aldrich, Diegem, Belgium) and put at −80 °C during 40 min and freeze-dried overnight with a freeze-dryer Epsilon 1-6D (Martin Christ, Osterode am Harz, Germany). Freeze-dried fungi were then beat-beaten with a Mini bead beater (Biospec Products, OK, USA) during 1 min at maximal speed.

Subsequently, the total DNA was extracted with an adapted phenol chloroform (24:1) protocol (Ashktorab and Cohen, 1992) and purified with the Qiagen CTAB genomic Tip-20 kit (Qiagen Benelux—B.V., KJ Venlo, The Netherlands) according to the manufacturer’s protocol. A 100-μl Gibco® DNase, RNase, protease free water (Life Technologies, Gent, Belgium) was used to elute the DNA. The DNA integrity was verified on a 2 % agarose gel. The DNA concentration and purity were evaluated with a Nanodrop® 2000 (Thermo Scientific, Wilmington, USA).

### Primer design

First, a collection of publicly available 18S rDNA sequences from *E. jeanslemei* strains and other closely related species (namely *E. lecanii-corni* and *E. spinifera*) and from the common water contaminant *A. fumigatus* (Heinemann et al. 1994; Parat et al. 1996), was made (NCBI, GenBank). The following sequences were included: for *E. jeanselmei*: AB531492.1/AF549447.1/AF050271.1/AJ866273.1/AY857530.1/AY163553.1/AY163549.1/AY163550.1 AY163552.1/AY163556.1/DQ836791.1/DQ836793.1/DQ836795.1/JN625228.1/JX192603.1/JX473278.1/EF025410.1/EF025411.1/EF025412.1/EU910261.1/JX473276.1; for *A. fumigatus*: KC411924.1/KC237295.1/KC237291.1/KC237292.1/KC142152.1/HE864321.1/KC119199.1/KC119200.1/JX944178.1/JX944118.1; for *E. lecanii-corni*: GQ426959.1/GQ426975.1/GQ426980.1/JN675374.1/JN675375.1/JX473283.1/JX473285.1/JX681038.1/JX681039.1/JX681040; for *E. spinifera*: AB025891.1/AB025892.1/AB025857.1/AB025876.1/AY156966.2/AY156970.1/EF551539.1/EF551459.1/KC952672.1/NR_111131.1. These sequences were aligned with the *MegAlign* software V10.0.1 (Lasergene, Madison, USA) to identify the ITS 1 and ITS 2 sequence regions of interest wherein different primer pairs were designed using the *Primer 3V.0.4* software (http://bioinfo.ut.ee/primer3-0.4.0/) (Untergasser et al. 2007). Primer dimers and secondary structure formation was evaluated and predicted during the design with Primer3. The *wprimersearch* software (https://wemboss.uio.no/wEMBOSS/) (Sarachu and Colet 2005) was used to perform an in silico specificity test, allowing to select the primer pair that only amplifies the target sequences. Also, BLASTn (http://blast.ncbi.nlm.nih.gov/Blast.cgi) was used to evaluate the specificity of the primers.

### Qualitative SYBR®green qPCR assay

The qPCR assay for the detection of *E. jeanselmei* (*Ejeanselmei*_*ITS* qPCR method) was performed as previously described for *A. versicolor* (Libert et al. 2015), using the SYBR®green chemistry and a real-time PCR IQ5 ™ system (Biorad, Temse, Belgium). All the primers used in this study were synthetized by Eurogentec (Liège, Belgium).

Briefly, the reaction mix (25 μl final volume) contained 12.5 μl of 2× SYBR®green PCR Mastermix (Diagenode, Liège, Belgium), 0.25 μl of *Ej*_*ITS* forward and reverse primers (0.2 μM) (Table 2) and 7 μl of Gibco® DNase, RNase, protease free water (Life Technologies, Gent, Belgium). To this mix, 5 μl of genomic DNA (gDNA) at 200 theoretical genomic copy numbers per microliter was added. The number of genomic DNA copies was calculated according to the formula presented below i.e.,

$$ {C}_{\mathrm{n}}=\frac{m\times {A}_{\mathrm{c}}}{M_{\mathrm{w}}\times {G}_{\mathrm{s}}} $$

**Table 2:** Primer sequences developed in silico

Name	Purpose	Sequence 5′ to 3′	Amplicon size (bp)
**Ej_ITS_f**	**Forward primer**	**ccgagttagggtcctcaca**	**70**
**Ej_ITS_r**	**Reverse primer**	**ggcctaccgaagcaacata**	
Ej_ITS_2f	Forward primer	cccggtacactgagcatctt	107
Ej_ITS_2r	Reverse primer	cctacctgatccgaggtcaa	

The amplicon size is expressed in base pairs (bp). In bold, the primers selected as efficient primer couple in the preliminary specificity test for the detection of *E. jeanselmei* (IHEM 4740)

with *C*_*n*_ = genomic copy number; *m* = the amount of gDNA (grams) and determined by Nanodrop®2000 (Thermo Scientific, Wilmington, USA); *Ac* the Avogadro’s constant (Mohr et al. 2008). *M*_*w*_ = base pair mean molecular weight (649 Da) and *G*_*s*_ = Genome size (expressed in bp) of *E. jeanselmei* = 30,000,000. Because no information on the genome size of *E. jeanselmei* is currently publicly available, we used an estimation based on the average of the genome size of *E. dermatitidis*, *E. xenobiotica*, *E. spinifera* (Broad Institute 2015). We are also aware that some strain-dependent deviations may exist.

The optimization of the qPCR conditions was performed with the *E. jeanselmei* strain BCCM/IHEM 4740. Therefore, this species was considered as a reference and used as a positive control added in each run performed in this study.

In each run, a *no template control* (NTC) was included for the analysis whereby the DNA template was replaced by ultrapure water in the reaction mix. This NTC aimed at verifying that no contamination occurred and that no primer dimers were formed.

The following thermal cycling conditions were used for all runs: 1 cycle of 95 °C for 10 min (Taq activation), followed by 40 amplification cycles of 15 s at 95 °C (denaturing step) and 60 °c for 1 min (annealing and extension step). Subsequently, a melting curve was made with a gradual increase of temperature of 0.5 °C/6 s from 55 to 95 °C during 15 min. The Biorad IQ 5 software V. 2 (Biorad, Temse, Belgium) automatically determined the threshold level for the reaction.

### Inhibition test (pure cultures)

Because PCR inhibitors (e.g., co-extracted substances or RNA contaminations) could affect the amplification, the validation results and also the detection of low amount of the targeted DNA, it is important to verify that all of these substances were removed during the DNA extraction step. This was done using an inhibition test. The workflow of this inhibition test was based on that proposed in 2012 by Broeders et al. for the assessment of absence of inhibitors in DNA extracts. Briefly, gDNA was extracted independently from two pure cultures of *E. jeanslemei* strain BCCM/IHEM 4740. Afterwards, a calibration curve was made based on the analysis of each set of gDNA, diluted 10, 100, 1000, and 5000 fold, with the *Ejeanselmei*_*ITS* SYBR®green assay. Two criteria exist to assess the absence of inhibitors in the DNA extracts; i.e., the slope of the calibration curve which should be between −3.6 and −3.1; and the difference (Δ *C*_q_) observed between the experimentally obtained *C*_q_ values and an extrapolated C_q_ obtained by the regression of the C_q_ from the undiluted sample (Broeders et al. 2012). In the absence of inhibition, the Δ *C*_q_ should be ≤0.50 for each dilution (Broeders et al. 2012, European Network of GMO Laboratories 2015).

### Sequencing

The identity of the strains of *E. jeanselmei* IHEM 4740, IHEM 4741, IHEM 22665, and of *E. spinifera* IHEM 20752 that resulted in a specific amplicon in the qPCR assay was confirmed based on the sequence of their ITS 1 and ITS 2 regions. Hereto, a dideoxy sequence analysis with the BigDye Terminator v3.1 cycle sequencing kit (Applied Biosystems, Life Technologies, Gent, Belgium) and an ABI3130xl Genetic Analyzer apparatus (Applied Biosystems, Life Technologies, Gent, Belgium) was used according to the manufacturer’s recommendations. Primers ITS1F (Gardes and Bruns 1993) and ITS4 (White et al. 1990) were first used to amplify the ITS 1 and ITS 2 regions. Primers ITS1 and ITS2 (White et al. 1990) were used for the subsequent sequencing reaction. The consensus sequences of each of the targeted regions (based on the forward and reverse sequence of each target region) were aligned with the MEGA v6.06 (http://www.megasoftware.net/) (Tamura et al. 2013) software and visualized with the CLC sequence viewer v7.0.2 (Qiagen Benelux—B.V., KJ Venlo, The Netherlands). Using BLASTn (http://blast.ncbi.nlm.nih.gov/), the amplification of the targeted DNA regions and their identity was confirmed by comparing them to the sequences available in the NCBI database.

### Theoretical *T*_m_ calculation

The online tool from IDT (http://eu.idtdna.com/calc/analyzer) (IDT, Leuven, Belgium) with the consensus sequences obtained through sequencing as input and under the PCR conditions described above, was used to in silico calculate the theoretical *T*_m_ of the amplicon obtained in the *Ejeanselmei*_*ITS* assay.

### *Ejeanselmei*_*ITS* assay performance assessment

For the performance assessment of the qPCR assay, the approach as previously outlined by Libert et al. (2015), which on its own is based on the study of Barbau-Piednoir et al. (2013), was followed. Different parameters of the qPCR method were evaluated, i.e., the selectivity (based on inclusivity and exclusivity), the PCR efficiency, the limit of detection (sensitivity test), and the repeatability.

#### Selectivity test

The selectivity test was composed of a preliminary and a larger selectivity test.

For the preliminary selectivity test, the target species (*E. jeanselmei* IHEM 4740) and one non-target species (*E. dermatitidis* IHEM 9780) were used, both at 15,000 theoretical copies of gDNA. The amplicon obtained for the *E. jeanselmei* strain IHEM 4740, which was taken as a reference in the performance assessment study, was confirmed by sequencing analysis.

The larger selectivity test aimed at evaluating the inclusivity (i.e., the selected primers should amplify the DNA of each tested strain from the target species) and the exclusivity (i.e., DNA of non-target species close to the target or described to frequently occur in the same environment as the target species should not be amplified by the selected primers, with a specific *T*_m_) of the *Ejeanselmei*_*ITS* qPCR method, using the selected primers and the qPCR conditions as described above. So, a result has been considered as positive when the sample is amplified and the observed *T*_m_ corresponds to the *T*_m_ defined in silico for the target. If these two conditions are not observed, the result has been considered as negative.

For the inclusivity, three target strains (i.e., *E. jeanselmei*) from the BCCM/IHEM collection were selected. The experimental design of the exclusivity test included 14 non-target strains i.e., 5 species closely related to *E. jeanselmei* (i.e., *E. dermatitidis*, *E. exophialae*, *E. lecanii-corni*, *E. spinifera* and *E. xenobiotica*) (Zeng and De Hoog 2008) and 9 other common species in wet environment as described by Heinemann et al. (1994) (i.e., *A. strictum*, *A. alternata*, *A. fumigatus*, *C. cladosporioides*, *C. herbarum*, *C. sphaerospermum*, *P. chrysogenum*, *S. chartarum* and *U. botrytis*)*.*

The conditions described above were used for each qPCR run using a total of 1000 theoretical copies of gDNA per reaction (evaluated for each target with its own corresponding genome size).

Based on the selectivity test, the false positives ratio (FPR) and false negatives ratio (FNR), the sensitivity and the selectivity values (%) were calculated according to the formulas presented by Blakely and Salmond (2002), i.e.,

$$ FPR=\mathrm{False}\ \mathrm{positives}/\left(\mathrm{False}\ \mathrm{positives}+\mathrm{True}\ \mathrm{negatives}\right) $$

$$ FNR=\mathrm{False}\ \mathrm{negative}/\left(\mathrm{False}\ \mathrm{negative}\mathrm{s}+\mathrm{True}\ \mathrm{positives}\right) $$

$$ \mathrm{Sensitivity}=\mathrm{True}\ \mathrm{positives}/\left(\mathrm{True}\ \mathrm{positives}+\mathrm{False}\ \mathrm{positives}\right)\times 100 $$

$$ \mathrm{Selectivity}=\mathrm{True}\ \mathrm{negatives}/\left(\mathrm{True}\ \mathrm{negatives}+\mathrm{False}\ \mathrm{negatives}\right)\times 100 $$

#### PCR efficiency estimation

The qPCR analysis of a serial dilution, in duplicate, of gDNA (1000, 500, 100, 50, 10, 5, 2, and 1 theoretical copy number of gDNA obtained by two independent extractions) of *E. jeanselmei* IHEM 4740 was used to assess the linearity of the SYBR®green qPCR assay. Based on this analysis, two parameters can be evaluated, i.e., the coefficient of determination (*R*^2^) and the PCR efficiency. *R*^2^ is an indicator of the correlation of the data regarding the linear regression curve. The PCR efficiency (*E*) calculation was previously described by Rutledge and Cote (2003). As previously described for *A. versicolor* (Libert et al. 2015), according to the most recent guidelines developed for GMO detection with qPCR SYBR®green (European Network of GMO Laboratories 2015), the *R*^2^ and amplification efficiency are not applicable to qualitative methods. However, a *R*^*2*^ ≥ 0.98 and a PCR efficiency ranging between 80 and 120 % have previously been indicated as performance criteria for the validation of qualitative qPCR methods (Broeders et al. 2014).

#### Limit of detection (sensitivity test)

The limit of detection (LOD) is defined as the lowest concentration of an analyte which is detected with a probability of 95 % (Barbau-Piednoir et al. 2013; Broeders et al. 2014). Six dilutions (i.e., 100, 50, 10, 5, 2, 1, 0.5, 0.2 and 0.1 theoretical copies of gDNA) of genomic DNA of *E. jeanselmei* IHEM 4740 were tested in six independent runs (using the qPCR conditions described above), each with six repetitions, to estimate the LOD of the *Ejeanselmei*_*ITS* assay. The LOD should be below 25 copies according to definition of minimum performance requirements for analytical methods of GMO testing (European Network of GMO Laboratories 2015; Libert et al. 2015).

#### PCR repeatability

This repeatability limit (*r*) is defined as the maximal difference of two results obtained under identical experimental conditions with a probability of 95 % (Barbau-Piednoir et al. 2013). The experimental design used for the LOD evaluation (see above) was also applied for the evaluation of the *r* value of the *Ejeanselmei*_*ITS* qPCR method.

As described previously (Barbau-Piednoir et al. 2013; Libert et al. 2015), the relative standard deviation of the repeatability (RSDr) was calculated as the absolute value of the coefficient variation (%). For these criteria, there is no limit fixed for qualitative qPCR methods (European Network of GMO Laboratories 2015). The RSDr, evaluated for the *C*_q_ values, should be below 25 % for all dilutions above the LOD for quantitative methods (Barbau-Piednoir et al. 2013; Broeders et al. 2014).

### Proof of concept: environmental testing

#### Sampling

The sampling method for air-conditioning systems was previously described by Nolard et al. (2004). Briefly, 1 l of water was collected in a sterile Duran bottle with a vacuum pump at a distance between 1 and 5 cm of the bottom of the tank of the air-conditioning system. In total, eight tanks from different air-conditioning systems were sampled. The water samples were stored at 4 °C until their analysis.

#### Classical analysis: culture, microscopic analysis and cell counting

Under laminar flow, a first part of the water sample was diluted 10 and 100 fold and each dilution, as also an undiluted aliquot of 1 ml, was poured into an empty petri dish. Afterwards, malt extract agar (MEA) chloramphenicol liquid medium (Biorad, Temse, Belgium) was put in the petri dish and the plate was kept at room temperature till the medium had solidified. Then, the plate was incubated at 25 °C for 21 days. A first microscopic analysis was made after 5 days of incubation in order to determine the fastest growing species and a second after 21 days of incubation to determine other species such as the *Exophiala* species.

#### Molecular analysis: DNA extraction and qPCR analysis

Additionally, aliquots of 15 ml were taken of mixed water samples used for the classical analysis described above. After 15 min of centrifugation at 5000×*g*, DNA from aliquots was extracted with the DNA extraction protocol used for pure cultures (see above). The DNA concentration and purity was evaluated with a Nanodrop® 2000 (Thermo Scientific, Wilmington, USA). The qPCR reactions were performed on 5 μl of eluted DNA i.e., 10 % of the total DNA eluted from water samples corresponding to the amount of DNA mentioned in the Table 5.

#### PCR inhibition test (spike test)

To verify that no inhibition from the anti-fungal chemistry used to clean the air-conditioning occurred on the PCR amplification, as part of these chemicals might have been retained in the DNA extract, 1000 theoretical copy numbers of *E. jeanselmei* gDNA (IHEM 4740) were spiked into 5 μl of DNA extract from sample 3 (considered as an *environmental* no template control, as by classical analysis, no *E. jeanselmei* was detected) and analyzed with the *Ejeanselmei*_*ITS* qPCR assay. To determine whether inhibition occurred during the qPCR reaction, the obtained *C*_q_ value with the spiked sample was compared to the *C*_q_ obtained with 1000 copy numbers of gDNA of *E. jeanselmei* IHEM 4740 in pure water (Table 1).

## Results

### Design and selection of qPCR primer pair

**Fig. 1:**
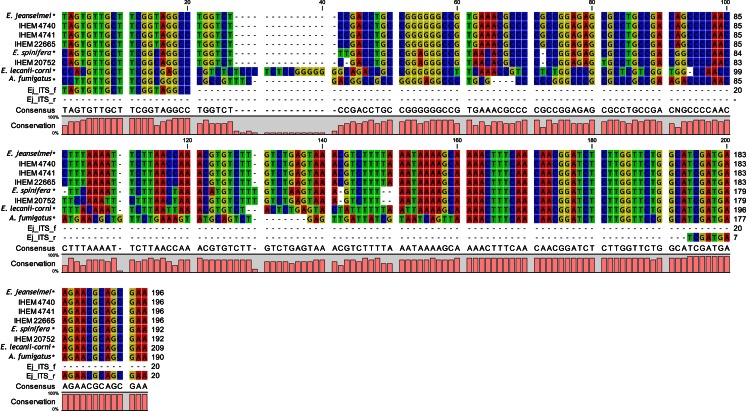
Alignment of region of ITS sequences of *E. jeanselmei*, *E. lecanii-corni*, *E. spinifera* and *A. fumigatus* strains, and of selected primers This alignment was made using publicly available ITS sequences of *E. jeanselmei*, *E. lecanii-corni*, *E. spinifera* and *A. fumigatus* extended with ITS sequences from the strains from the BCCM/IHEM collection used during the performance assessment of the qPCR assay (indicated with IHEM prefix) and with the primers designed in this study (*Ej*_*ITS*_f and *Ej*_*ITS*_r). Because no nucleotide variation was detected for each of the public sequences used, only one sequence (*) was introduced for this alignment, as a representative for that species. Consensus (last line of the alignment) corresponds to a consensus sequence defined by the software. The conservation level among each sequence (0 to 100 % of conservation) is represented by the pink rectangles at the bottom of the figure. The alignment was made with MEGA 6.06 software (http://www.megasoftware.net/) and visualized with CLC sequence viewer 7 (Qiagen Benelux—B.V., KJ Venlo, The Netherlands)

For the design of the *E. jeanselmei-*specific qPCR primers, all the *E. jeanselmei* 18S rDNA sequences that were publicly available at that time were used. This selection of sequences was extended with those available for the closely related species *E. lecanii-corni* and *E. spinifera*, which are belonging together with *E. jeanselmei* to the *E. spinifera* clade (Wang et al. 2001; Zeng and De Hoog 2008), and those available for *A. fumigatus*, a common species observed in water (Heinemann et al. 1994; Parat et al. 1996). Based on an alignment of these sequences, two couples of *E. jeanselmei* primers targeting a conserved region within the ITS 2 region of the 18S rDNA of *E. jeanselmei* were designed (Table 2, Fig. 1). It was however not possible to design primers in this region that were exclusively specific to *E. jeanselmei*, as part of the primers were also conserved in *E. spinifera*, with some nucleotide variations especially in the forward primer annealing site. The impact of this sequence conservation was addressed during the specificity test as part of the performance assessment (see below).

Preliminary specificity tests with these two primer couples were performed on the ITS 2 regions of *E. jeanselmei* (IHEM 4740) and *E. dermatitidis* (IHEM 9780) as a negative control, showing that only the ITS 2 region of *E. jeanselmei* was amplified (data not shown). Finally, based on the best combination of primers in terms of amplification efficiency (data not shown), the *Ej*_*ITS*_f and *Ej*_*ITS* r primers (Table 2) were selected for the detection of *E. jeanselmei* in this qPCR assay (*Ejeanselmei*_*ITS* assay).

**Fig. 2:**
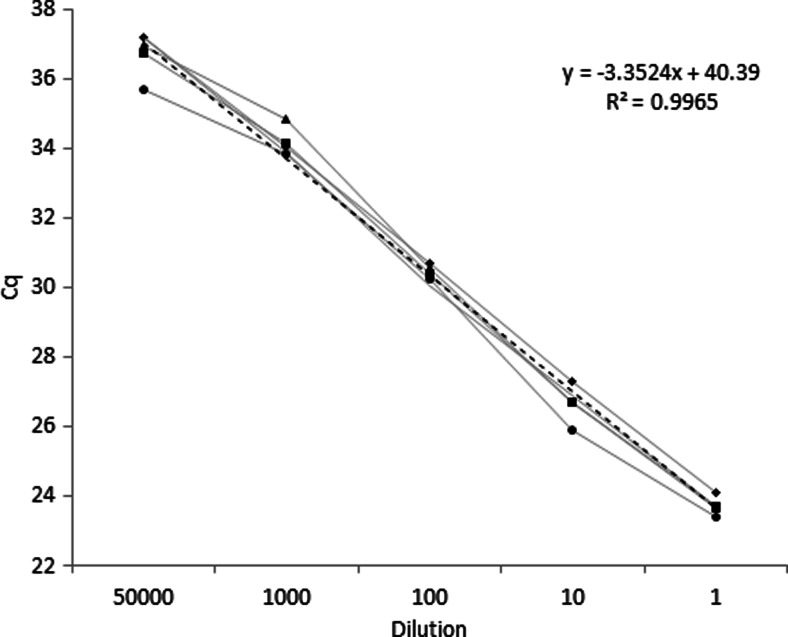
Calibration curve (inhibition test). Data were obtained with four replicates of four dilutions (5000, 1000, 100, 10 fold) of gDNA of *E. jeanselmei* (IHEM 4740). The *dotted line* corresponds to the trend line

In order to verify that no inhibition occurred during the PCR amplification, an inhibition test was performed with the selected primers. The slope of the calibration curve obtained with the two sets of gDNA dilutions was determined to be −3.35 (Fig. 2). This slope is in the range of −3.1 to −3.6 as recommended by the ENGL (2011). Δ *C*_q_ values were calculated for each dilution, i.e., 0.27 for the 5000-fold dilution, 0.43 for the 1000-fold dilution, 0.02 for the 100-fold dilution and 0.33 for the 10-fold dilution. According to the acceptance criteria from the ENGL for GMO for the assessment of the absence of inhibitors in the DNA extracts (ENGL 2011), no inhibition occurred.

### *Ejeanselmei*_*ITS* assay performance assessment

To evaluate the quality of this *Ejeanselmei*_*ITS* assay, a performance assessment was done as previously described for *A. versicolor* (Libert et al. 2015), according to the guidelines defined for the validation of qPCR detection and identification methods in other fields (Barbau-Piednoir et al. 2013; Broeders et al. 2014). The following criteria were evaluated: the selectivity of the primers, the LOD, the PCR efficiency, the dynamic range, and the *Ejeanselmei*_*ITS* assay repeatability.

#### Selectivity of the *Ejeanselmei_ITS* qPCR assay

**Fig. 3:**
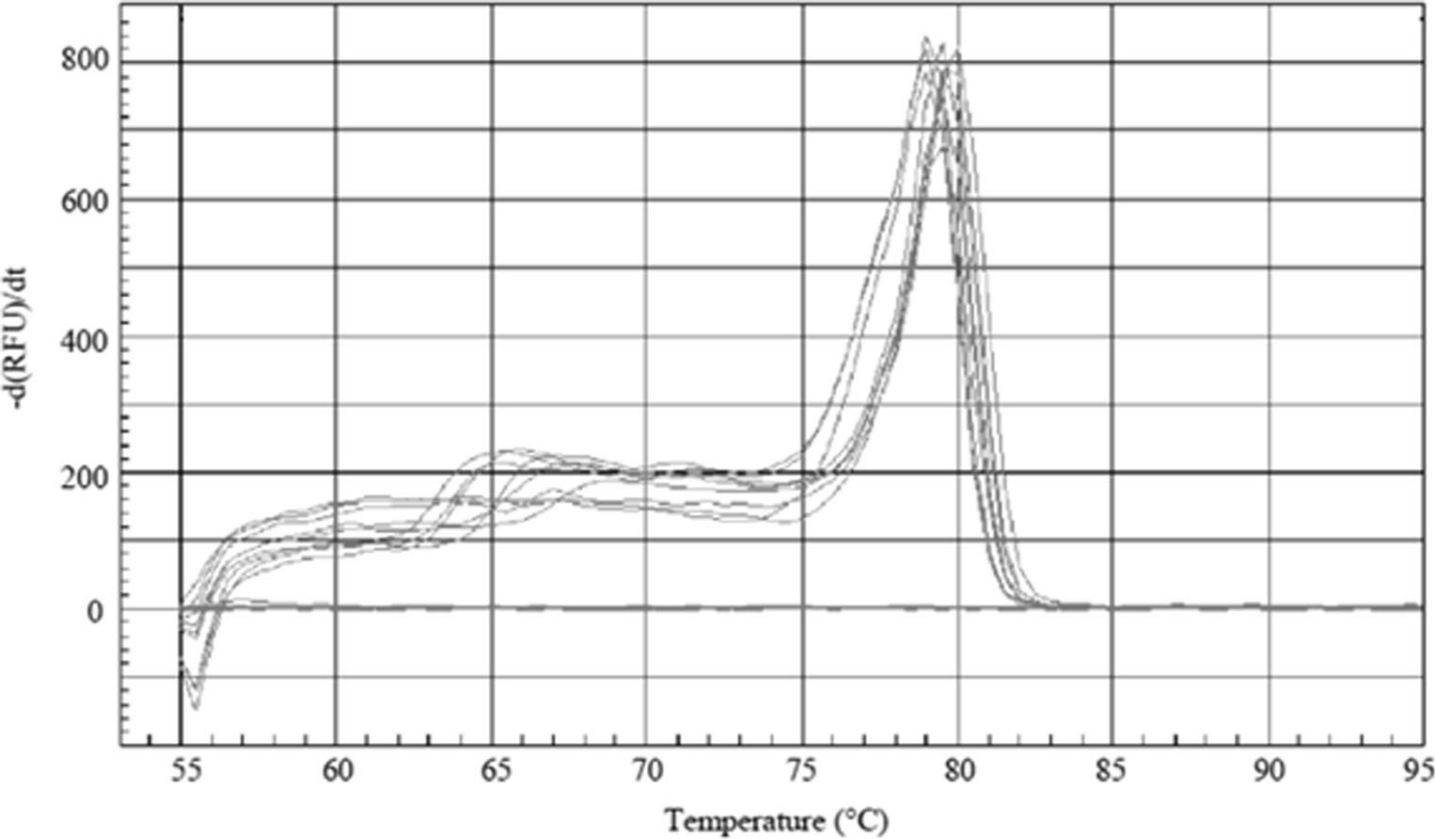
Melting curves obtained with the *Ejeanselmei*_*ITS* qPCR assay for the *E. jeanselmei* pure strains listed in Table 1. The melt curves were obtained with the Biorad IQ 5 software V. 2 (Biorad, Temse, Belgium). The *X-axis* shows the temperature (°C). The *Y-axis* presents the inverse of the first derivate of the best-fitted curve of the measured fluorescence decrease. The *gray curves* correspond to the *E. jeanselmei* listed in Table 1. The *blue flat curves* represent the NTC

First, an inclusivity test was performed with DNA of 3 *E. jeanselmei* strains from the BCCM/IHEM collection. We have included all in this collection available *E. jeanselmei* strains originating from water. DNA of each of the selected strains (*E. jeanselmei* BCCM/IHEM 4740, BCCM/IHEM 4741, BCCM/IHEM 22665) was amplified (3/3) with a *C*_q_ of, respectively, 20.86 ± 0.22, 21.81 ± 0.43 and 23.57 ± 0.38 for 1000 copies of gDNA (Table 1). The melting curve analyses showed *T*_m_ values between 79.25 ± 0.50 and 79.75 ± 0.25 (Table 1, Fig. 3).

The sequence of these BCCM/IHEM strains matches perfectly with the ones publicly available for the corresponding region and all amplicons showed 100 % identity (Fig. 1). The obtained *T*_m_ for each amplicon corresponds to the theoretical *T*_m_, which was calculated to be 79.50 °C. No false negatives were obtained.

Then, an exclusivity test was performed on DNA of non-target species (i.e., *A. strictum*, *A. alternata*, *A. fumigatus*, *C. cladosporioides*, *C. herbarum*, *C. sphaerospermum*, *E. dermatitidis*, *E. exophialae*, *E. lecanii-corni*, *E. spinifera*, *E. xenobiotica*, *P. chrysogenum*, *S. chartarum* and *U. botrytis*)*.* These species are closely related to *E. jeanselmei* and/or are occurring in the same environment (i.e., water reservoir) and/or in indoor environment (Al-gabr et al. 2014; Anaissie et al. 2003; De Hoog et al. 2003; Heinemann et al. 1994; Kawasaki et al. 1999). No false positives were observed (Table 1). Indeed, with this *Ejeanselmei*_*ITS* qPCR assay, except for DNA extracted from the *E. spinifera* IHEM 20752 strain, no DNA from non-targeted species was amplified. The amplification of *E. spinifera* was not unexpected based on the in silico analysis including the alignment (Fig. 1), which included all the publicly available ITS sequences of *E. jeanselmei* and *E. spinifera*. Because the sequence between the two primers was found to be identical for all the sequences available for one species, only one sequence for each species has been represented in the figure.

**Fig. 4:**
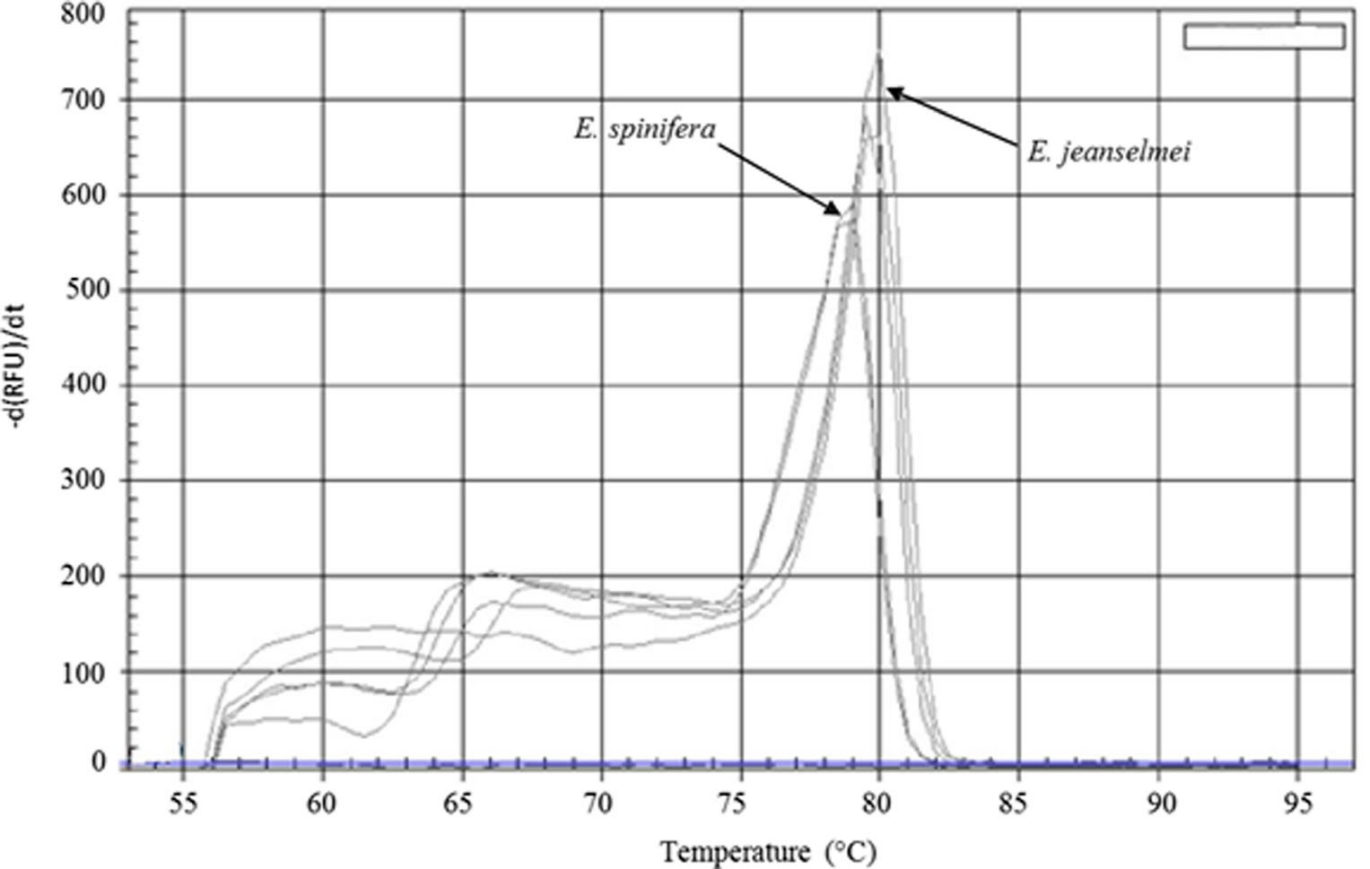
Melting curves obtained with the *Ejeanselmei*_*ITS* qPCR assay for the *E. jeanselmei* and *E. spinifera* pure strains listed in Table 1. The melt curves were obtained with the Biorad IQ 5 software V. 2 (Biorad, Temse, Belgium). The *X-axis* shows the temperature (°C). The *Y-axis* presents the inverse of the first derivative of the best-fitted curve of the measured fluorescence decrease. The *blue flat curves* represent the NTC

However, the *E. spinifera* amplification should be considered as a true negative results based on the SYBR®green characteristic. Indeed, for a same copy number estimation (i.e., 1000 theoretical genomic copy number), the obtained C_q_ value for *E. spinifera* was 26.04 ± 0.05 (Table 1) and the *T*_m_ was 78.50 °C (Table 1, Fig. 4), which are different from the ones obtained for *E. jeanselmei*. Based on these amplification results and *T*_m_ values, no false negative values were observed, i.e., FPR and FNR values of 0 % were obtained.

To explain these differences, amplicons of *E. jeanselmei* and *E. spinifera* were sequenced and aligned (Fig. 1). The amplicon of *E. spinifera* differs from that obtained for *E. jeanselmei* with 11 nucleotides, of which 2 in the annealing site of the forward primer (Fig. 1). These results were expected and met those obtained during the primer design (Fig. 1). These nucleotide differences explained the difference in the observed *T*_m_ for the two species (Table 1). The BLAST analysis of the ITS 1 and ITS 2 regions confirmed the IHEM 20752 as *E. spinifera* with 97 % of identity.

Based on these results, a sensitivity of 100 % and a specificity of 100 % were observed. An NTC was included in each assay to verify that no contamination occurred during the qPCR assay preparation (preparation of the mixes, filling of the qPCR plates). In none of the assays, the NTC resulted in a signal. This also showed that no dimerization of primers occurred during the analysis, as predicted during the in silico test (Fig. 3).

#### Limit of detection (sensitivity test) and PCR repeatability

The limit of detection (LOD) of the *Ejeanselmei*_*ITS* assay, based on 6 independent runs with a total of 18 repetitions, was determined to be one theoretical copy number (Tables 3 and 4) (*C*_q_ = 34.86 ± 0.90). The *r* and RSDr were 3.45 and 9.73 % respectively for this assay.

**Table 3:** *C*_q_ values obtained during the six runs of the limit of detection estimation for qPCR SYBR®Green assay *Ejeanslemei*_*ITS*

	**Run 1**						**Run 2**						**Run 3**					
Theoretical (estimated) copy number of gDNA	Rep. 1		Rep. 2		Rep. 3		Rep. 4		Rep. 5		Rep. 6		Rep. 7		Rep. 8		Rep. 9	
10	33.47	32.63	32.55	32.55	32.42	32.93	32.03	31.65	31.81	31.38	31.58	31.78	30.73	30.41	29.98	30.43	30.14	30.42
5	33.91	33.88	33.91	34.02	34.16	34.07	32.78	32.74	32.07	32.06	32.43	32.82	31.74	31.55	31.58	30.82	31.07	31.12
2	36.50	35.16	35.03	35.05	35.35	35.18	33.20	33.37	33.38	33.78	33.20	33.65	32.84	32.75	32.70	32.33	33.15	33.78
1	36.89	36.27	35.54	35.87	36.38	36.18	35.67	33.84	33.77	34.43	36.13	34.59	33.92	34.12	34.17	33.81	34.07	34.07
0.5	37.98	36.61	37.92	37.46	37.79	36.55	35.64	35.94	35.81	36.08	36.66	36.09	34.35	35.09	34.61	35.14	34.40	36.31
0.2	N/A	37.59	38.21	37.91	38.50	38.72	37.01	N/A	N/A	36.69	37.97	37.31	35.95	N/A	35.94	36.22	36.51	N/A
0.1	N/A	39.59	N/A	39.44	N/A	39.28	38.17	N/A	38.66	N/A	N/A	36.81	N/A	N/A	N/A	N/A	N/A	N/A
	**Run 4**						**Run 5**						**Run 6**					
*Theoretical copy number of gDNA*	*Rep. 10*		*Rep. 11*		*Rep. 12*		*Rep. 13*		*Rep. 14*		*Rep. 15*		*Rep. 16*		*Rep. 17*		*Rep. 18*	
10	31.76	31.68	31.79	32.05	31.55	31.82	30.87	31.07	31.03	30.96	31.00	31.34	31.61	31.59	31.30	31.28	31.09	31.70
5	32.71	32.07	32.33	32.70	32.83	32.77	31.76	31.72	32.06	31.97	31.63	31.98	33.26	32.31	32.16	32.63	32.06	32.43
2	34.15	33.17	33.35	33.51	33.85	34.50	32.57	32.89	33.52	32.48	32.64	32.78	34.75	34.72	33.99	34.53	34.09	34.18
1	34.33	34.33	36.08	34.96	34.46	34.03	35.45	33.86	33.66	34.76	35.29	34.20	35.12	N/A	34.62	35.44	34.48	35.19
0.5	37.83	36.17	36.67	35.50	35.91	36.24	36.56	35.33	37.09	35.43	34.69	35.88	38.39	37.13	N/A	N/A	37.13	36.71
0.2	37.64	38.06	39.55	N/A	38.91	38.02	37.30	36.07	35.88	36.91	38.14	37.75	38.31	38.32	38.91	39.55	37.08	N/A
0.1	N/A	36.61	N/A	N/A	N/A	N/A	N/A	39.61	37.34	N/A	N/A	N/A	N/A	N/A	N/A	N/A	N/A	N/A

Mean of *C*_q_ value obtained for six repetitions (Repetition [*Rep.*] 1 to 18) of 6 independent runs (Run 1 to 6) of a serial dilution of genomic DNA of *E. jeanselmei* (concentration expressed in copy number of genomes of the IHEM 4740 strain). The LOD is defined by the dashed line

**Table 4:** Limit of detection results for *Ejeanselmei*_*ITS* qPCR SYBR®Green assay

Theoretical (estimated) copy number	*C*_q_ mean ± SD	% positive
10	31.51 ± 0.78	100.00 (36/36)
5	32.45 ± 0.88	100.00 (36/36)
2	33.78 ± 0.98	100.00 (36/36)
1	34.86 ± 0.90	97.22 (35/36)
0.5	36.27 ± 1.07	94.44 (34/36)
0.2	37.62 ± 1.06	80.56 (29/36)
0.1	38.39 ± 1.21	25.00 (9/36)

The table shows the mean *C*_q_ value obtained for six repetitions of six runs of a serial dilution of genomic DNA of *E. jeanselmei* (concentration expressed in copy number), the standard deviation (±SD) and the percentage of positive response observed at each dilution point. The LOD is defined by the discontinuous line

#### Dynamic range and PCR efficiency

**Fig. 5:**
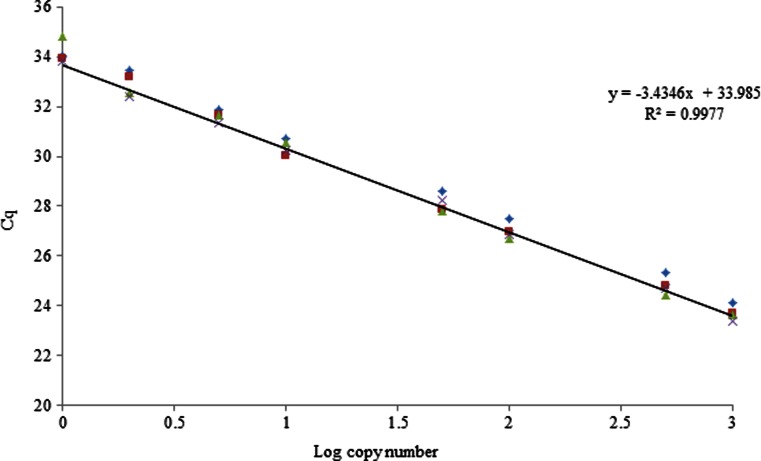
Coefficient of determination and PCR efficiency of *Ejeanselmei*_*ITS* qPCR assay Data are obtained with four replicates for each concentration i.e., 1000 to 0.1 theoretical copy number of genome of *E. jeanselmei* (IHEM 4740)

A serial dilution of 1000 to 0.1 theoretical genomic copy numbers of *E. jeanselmei* permitted to define the dynamic range and PCR efficiency of the *Ejeanselmei*_*ITS* assay. A linear model with a *R*^2^ of 0.9977 and an efficiency of 95.5 % were obtained with this SYBR®green assay (Fig. 5).

### Environmental testing

To evaluate the performance of this *E. jeanselmei*_*ITS* method on real-life samples, a test was performed on different water samples collected from air-cooled systems in office buildings. This proof of concept allows to test the *Ejeanselmei*_*ITS* assay on environmental samples and to compare this method of detection with a classical routine analysis method (Table 5).

**Table 5:** Environmental testing on water from air-conditioning reservoirs

	Classical method		Molecular method		
Sample number	Species	CFU/ml ^a^	Amount of DNA/PCR reaction (ng)^b^	C_q_ mean ± SD ^c^	Theoretical copy number of gDNA for 1 ml ^d^
1	*E. jeanselmei*	2	2.93	35.20 ± 0.72	1
	Sterile mycelium	5			
2	*Acremonium* sp.	5	2.53	N/A	
	*Penicillium* sp.	1			
3	N/D	0	0.36	N/A	
4	*E. jeanselmei*	4	3.27	34.63 ± 0.76	2
	*Acremonium* sp.	2			
5	N/D	0	0.94	N/A	
6	*A. fumigatus*	1	1.73	N/A	
7	*E. jeanselmei*	3		35.24 ± 0.34	1
	*Acremonium* sp.	1	3.07		
	Sterile mycelium	1			
8	*Acremonium* sp.	17	9.80	N/A	

^a^ The value for CFU/ml is an estimation of the fungal contamination based on the number of colonies per plate.

^b^5 μl of the extracted DNA from 15 ml of sampled water (and eluted in 100 μl of extra pure DNAase, RNAase, protease free water) were used in a 25 μl-PCR reaction.

^c^ *C*_q_ values are C_q_ means (≤ 40) ± standard deviation (SD) obtained with the validated *Ejeanselmei*_*ITS* assay with 4 technical replicates.

^d^Theoretical copy number of gDNA based on the *E. jeanselmei* IHEM 4740 strain defined as the strain of reference of the performance assessment of this *Ejeanselmei*_*ITS* qPCR SYBR®green assay

The detection method used in routine (culture, counting, and microscopic identification) allowed to detect *E. jeanselmei* in three water samples with an amount ranging between 2 CFU/ml and 4 CFU/ml (Table 5). All of these samples contained one or two other contaminants frequently recovered from water reservoirs (Table 5) i.e., *Acremonium* sp., *A. fumigatus* and a sterile mycelium (undetermined).

The results from the *Ejeanselmei*_*ITS* assay were in accordance with those of the classical method for the detection of *E. jeanselmei*. Indeed, this qPCR assay gave positive signals for the same water samples where *E. jeanselmei* was detected using the classical methods, i.e., sample nos. 1, 2, and 3 (Table 5). The obtained *C*_q_ values were all ranging around 35 and the *T*_m_ values around 79.5 °C, as expected. No signal was observed for the samples where no *E. jeanselmei* was detected on plate. The spike test confirmed that no inhibition from potentially remaining anti-fungal chemistry products occurred during the qPCR analysis as a *C*_q_ of 22.50 ± 0.68 was obtained which corresponds to the value shown in Table 1 for *E. jeanselmei* IHEM 4740.

## Discussion

*E. jeanselmei* is frequently found inside buildings in canalizations, reservoirs of drinking water, non-drinking water reservoirs for air-conditioning. As this species is an opportunistic pathogen causing health problems (Al-gabr et al. 2014; Nucci et al. 2001; Nucci et al. 2002; Zeng et al. 2007), its monitoring is important. Classical approaches used for the detection of this pathogenic fungus are difficult because of morphologically closely related species and are moreover time consuming. Indeed *E. jeanselmei* requires up to 21 days of incubation at 35 °C before microscopic identification (Najafzadeh et al. 2013; Nishimura et al. 1987; Nolard et al. 2004). This is why molecular analysis, such as the in this study developed *Ejeanselmei*_*ITS* assay, could be attractive for a more efficient monitoring and diagnosis of a contamination by *E. jeanselmei*. The real-time PCR analysis, and especially the SYBR®green qPCR chemistry, offers the advantage to be fast, sensitive, instrumental-wise more accessible and more cost-effective than other molecular techniques like direct sequencing, especially when many samples need to be analyzed. Indeed, the SYBR®green chemistry is cheaper than the chemistry used for sequencing, with a cost of less than 1 euro per reaction. In comparison with the classical tools, this technique is more expensive. However, the time saving (2 days against 21 for the classical approach) is a key advantage in terms of monitoring and diagnosis.

Another advantage is that the specificity is based on a primer couple and on the associated *T*_m_ value of the generated amplicon, thereby avoiding a sequencing step which is needed when using classical PCR approaches (Klein, 2002). In this assay, the primers were designed in the ITS 2 region from the 18S rDNA complex.

Because no guidelines nor norms exist for the development of qPCR methods for the detection of fungi, the performance assessment flow was based on guidelines and recommendations given for GMO detection and foodborne pathogen (Barbau-Piednoir et al. 2013; Broeders et al. 2014), as was previously done for a qPCR assay for the specific detection of *A. versicolor* (Libert et al. 2015)*.* To evaluate the performance of this *Ejeanselmei*_*ITS* SYBR®green assay, the selectivity, PCR efficiency, dynamic range, sensitivity and repeatability parameters were investigated.

First, the inclusivity tests revealed that the *Ejeanselmei*_*ITS* assay detects all the tested *E. jeanselmei* strains. Nevertheless, between these two amplified species, a variation of approximately 3 *C*_q_ was observed. As shown by sequencing analysis (Fig. 1), no variation occurred between the amplicon obtained for each of these strains, and the sequence between the primers matched 100 % with the corresponding one retrieved for all publicly available *E. jeanselemi* ITS 2 sequences. This variation in the *C*_q_ could however be explained by a dissimilarity of the 18S rDNA copy number which is known to have an interspecies and an intraspecies variability (Black et al. 2013; Corradi et al. 2007; Iwen et al. 2002; Schoch et al. 2012), and which was also suggested for *A. versicolor* (Libert et al. 2015). Because no variation rates for the 18S rDNA copy number are known for *E. jeanselmei* or closely related species, this *Ejeanselmei*_*ITS* assay is a qualitative qPCR**.** To develop a quantitative tool based on the ITS sequence, the range of variation of the 18S rDNA copy number should be determined for each targeted strain in order to extrapolate the quantity expressed in genome copy numbers.

The exclusivity test, the second step of the specificity test, showed that no non-targeted strains from indoor environment, including the air-conditioning system, were detected with the *Ejeanselmei*_*ITS* assay. This demonstrates the good specificity of the *Ejeanselmei*_*ITS* assay. For none of the closely related and morphologically difficult-to-discriminate species was the DNA was amplified with the *Ejeanselmei*_*ITS* assay, except for *E. spinifera*. However, despite this amplification, the melting curve analysis allowed the discrimination between *E. spinifera* and *E. jeanselmei*, as the *T*_m_ differs with 1 °C between the two species (*T*_m_*E. spinifera* = 78.50 °C, *T*_m_*E. jeanselmei* = 79.50 °C) (Fig. 4). This difference in *T*_m_ can be explained by the interspecies diversity observed in the ITS region of species from the *E. spinifera* clade which includes *E. jeanselmei* (Wang et al. 2001; Woo et al. 2013a; Zeng and De Hoog 2008) (Fig. 1). The post-analysis based on the melting temperature to discriminate a species is currently used with the SYBR®green chemistry also in other fields to discriminate two different strains amplified by the same couple of primers (Barbau-Piednoir et al. 2013), without the need for sequencing confirmation.

In the future, high-resolution melting (applied to the *Ejeanselmei*_*ITS* assay) might be used to obtain an even more pronounced discrimination of these two *Exophiala* species based on the *T*_m_ of the amplicon, if needed. Nevertheless, the *Ejeanselmei*_*ITS* assay allows to discriminate between species that are frequently confused with *E. jeanselmei* with culture-dependent analysis and some molecular methods like classical PCR.

Then, efficiency and PCR linearity were evaluated for this SYBR®green assay. To evaluate these parameters, the strain *E. jeanselmei* IHEM 4740 was selected as the reference strain. Because the C_q_ values obtained for these strains were the highest among those for the strains tested, the results obtained for these parameters correspond to the worst case scenario for this assay. With an efficiency of 95.5 %, this SYBR®green assay is efficient according to the criteria defined for qualitative analysis of GMO (Broeders et al. 2014). Moreover, the obtained *R*^2^ value (0.9977) shows the linearity and the accuracy of this qPCR SYBR®green assay.

Furthermore, with an LOD defined at one theoretical genomic copies per reaction, this *Ejeanselmei*_*ITS* is sensitive and well within the criteria put forward by the GMO community (European Network of GMO Laboratories 2015). This *Ejeanselmei*_*ITS* assay is also repeatable with *r* (3.45) and *RSDr* (9.73 %) values below the limits defined by the guidelines used in this study (Barbau-Piednoir et al. 2013; Broeders et al. 2014). Because no information was available on the genome size of *E. jeanselmei,* the copy number estimation was done with an average of the genome size based on the size of three *Exophiala* species (i.e., *E. dermatitidis*, *E. spinifera*, and *E. xenobiotica*). Therefore, this estimation should be reviewed when the real genome size of *E. jeanselmei* will be available. Nevertheless, if the genome size of *E. dermatitidis* (i.e., 26.35 Mb) is used as *worst case* scenario to define the copy number of gDNA of *E. jeanselmei*, the underestimation of the copy number at LOD is evaluated to be 1 % only, which should not influence drastically our results.

These performance assessment tests were done using DNA extracted from pure strains from the BCCM/IHEM collection, which are well characterized. In order to test this *Ejeanselmei*_*ITS* assay under environmental conditions, water samples from air-conditioning were submitted to our qPCR method. The obtained results were compared with those obtained with the routine protocol based on the classical analysis method (i.e., culture, microscopic visualization, and counting). As similar results were obtained for both classical and qPCR method, this proof of concept demonstrated that the *Ejeanselmei*_*ITS* assay could be useful for the monitoring of *E. jeanslemei*. Concerning the time period to obtain these results, this *Ejeanselmei*_*ITS* assay seems to be a better alternative to the classical analysis. Indeed, using the classical methods, the incubation period was 21 days, while our SYBR®green analysis (lyophilization step and DNA extraction included) required 2 days. A low diversity, maximum two species per sample, was observed with the classical method as it was previously observed in other study (Hamada and Fujita 2002; Kelkar et al. 2005; Parat et al. 1996). In total, five species were observed (*Acremonium* sp., *A. fumigatus*, *E. jeanselmei*, *P. chrysogenum*) (Table 5). This implies that the water reservoirs were regularly cleaned with anti-fungal chemistry to avoid health problems such as allergies, asthma, or sick building syndrome. Therefore, it was verified that this anti-fungal chemicals did not inhibit the qPCR reaction, which was found not to be the case. Based on the *C*_q_ values obtained for the LOD estimation, an extrapolation of the DNA theoretical copy number was performed in order to compare the results from the classical and the molecular methods. According to this estimation, the theoretical (estimated) copy number of genomic DNA for *E. jeanselmei* was evaluated to 1 and 2 copy number of DNA per ml of analyzed water. These estimations were in the range of those obtained with classical methods (between 2 and 4 CFU/ml). However, some considerations should be made. Firstly, this extrapolation was done based on the values obtained for the reference strain (with the estimated genome size of 30 Mb), and it is not so unlikely that another strain of *E. jeanselmei* was present in the real-life samples. Therefore the effect of the variation in 18S rDNA copy number should be taken into account. Secondly, this comparison supposed that one CFU observed on plate corresponds to one copy of gDNA. However, a colony can originate from more than one copy of gDNA if aggregates were present. Therefore, although the range of detected *E. jeanselmei* contamination was comparable for both methods, and therefore the qPCR assay could be regarded as a semi-quantitative method, the absolute quantities of *E. jeanselmei* are difficult to be compared between the two analysis methods. However, this was expected, because as elaborated above, the ITS-based qPCR method is a qualitative method. If an absolute quantification is needed, the classical methods should still be used.

The results of this proof of concept demonstrate that molecular methods such as qPCR could also be used for the detection of other contaminants present in the water reservoir of air-conditioners. In order to reduce even more the analysis time, it should also be interesting to develop a multiplex tool for the detection of several contaminants simultaneously. This will allow achieving the same advantage of the classical method, which is not limited to the detection of *E. jeanselmei* only.

In addition, the use of this SYBR®green qPCR assay should not be reduced to water environments. It would also be useful for the detection of *E. jeanselmei* in other environmental or in clinical samples. As qPCR methods, being based on DNA amplification, offer the advantage of detecting also non-culturable/dead organisms, it would be interesting to apply this method to the monitoring of *E. jeanselmei* in environments where its occurrence has not yet been reported. Indeed, because *E. jeanselmei* requires a humid environment to be kept alive and the currently used monitoring methods for this pathogen are based on culturing (which implies that the to-be-detected organism should be alive and culturable), its presence in for instance indoor air, where the organism could suffer from desiccation impacting its growth, could not yet be demonstrated.

In conclusion, this paper reports on a novel SYBR®green qPCR assay for the specific detection of *E. jeanselmei,* called *Ejeanselmei*_*ITS*. Because classical methods are time consuming, and *E. jeanselmei* has demanding growth conditions, this qualitative assay, and molecular tools like qPCR in general, offers the possibility to reduce the analysis time period and to extend the monitoring to other environments. This will contribute to an improved response against fungal contamination and a better insight into the causal link between *E. jeanselmei* and health problems.
